# Functional connectivity between the uncinate fasciculus and frontotemporal semantic system supports reading comprehension in adolescents

**DOI:** 10.1162/IMAG.a.1055

**Published:** 2025-12-12

**Authors:** Tin Q. Nguyen, Emily M. Harriott, Yurui Gao, Andrea N. Burgess, Addie C. Cavender, Chenglin Lou, Kurt G. Schilling, Bennett A. Landman, John C. Gore, Laurie E. Cutting

**Affiliations:** Department of Special Education, Peabody College of Education and Human Development, Vanderbilt University, Nashville, TN, United States; Vanderbilt University Institute of Imaging Science, Vanderbilt University Medical Center, Nashville, TN, United States; Vanderbilt Brain Institute, Vanderbilt University, Nashville, TN, United States; Department of Biomedical Engineering, Vanderbilt University, Nashville, TN, United States; Department of Radiology and Radiological Sciences, Vanderbilt University Medical Center, Nashville, TN, United States; Department of Electrical and Computer Engineering, Vanderbilt University, Nashville, TN, United States; Vanderbilt Kennedy Center, Vanderbilt University Medical Center, Nashville, TN, United States

**Keywords:** white matter, gray matter, functional MRI, connectivity, reading

## Abstract

Skilled reading arises from the coordinated activity of neural systems supporting word recognition, semantic processing, and executive control. While the structural architecture of white matter tracts involved in reading is well characterized, their functional contributions remain unclear. Here, we examined whether resting-state functional connectivity of the left uncinate fasciculus, a ventral frontal-temporal white matter tract implicated in semantic processing, modulates the relationship between word recognition and reading comprehension. Fifty-three participants (ages 10–14 years; 29 girls, 24 boys) completed resting-state functional MRI and standardized assessments of word recognition and reading comprehension. Functional connectivity between the left uncinate fasciculus and structurally connected gray matter regions, including the anterior and medial temporal lobes and the ventrolateral prefrontal cortex, was derived from blood oxygen level-dependent (BOLD) signal correlations at rest. Regression analyses revealed that stronger uncinate fasciculus functional connectivity with semantic memory and control regions was associated with a weaker dependence of reading comprehension on word recognition skill. These findings suggest that semantic brain systems accessed via the uncinate fasciculus may support flexible meaning-based reading strategies, allowing comprehension to be sustained across varying levels of word recognition. By linking white matter functional connectivity to individual differences in reading performance, this study highlights the importance of semantic pathways in reading development and underscores the value of examining white matter–gray matter interactions in functional brain networks for reading.

## 1 Introduction

Skilled reading requires the seamless integration of various cognitive processes, including recognizing words, linking words to meanings, and integrating semantic information and background knowledge for comprehension (Castles et al., 2018; Kim, 2017). With advances in magnetic resonance imaging (MRI) technology, it is now possible to understand the functional and structural (including microstructural) properties of the brain that support the recruitment and coordination of different neural systems that support these higher-level, linguistically based cognitive processes. Although to date an extensive research body has characterized the activity of cortical gray matter regions involved in reading (Aboud et al., 2016; Bailey et al., 2018; Landi et al., 2013; Ozernov-Palchik et al., 2021; Roe et al., 2018; Ryherd et al., 2018), as well as the structural components of key white matter pathways (e.g., arcuate, superior longitudinal, inferior longitudinal fasciculi) (Del Tufo et al., 2019; Farah et al., 2022; Feldman et al., 2012; Meisler & Gabrieli, 2022; Roy et al., 2024), the *functional* characteristics of white matter pathways (fasciculi) that interconnect these regions remain largely underexplored. Establishing how the functional properties of white matter pathways support higher-level cognition is key for advancing our knowledge of neural circuitry associated with linguistic processing, including reading, in the brain. Here, we measure the relationship between functional properties of a key white matter tract related to linguistic processing—the uncinate fasciculus—and reading skills (Dick et al., 2014; Fridriksson et al., 2016; Friederici, 2011; Shekari & Nozari, 2023; Von Der Heide et al., 2013). Centrally, our findings demonstrate that functional properties of specific white matter fasciculi can be reliably linked to circumscribed cognitive processes.

White matter tracts serve as conduits for information transfer between gray matter regions. To date, these tracts have been primarily studied structurally via diffusion MRI (e.g., Assaf & Pasternak, 2008; Yeatman et al., 2013; Yendiki et al., 2011), limiting deep understanding of how information transfer occurs along white matter fasciculi functionally as signals travel between cortical regions. Until recently, capturing the functional properties of white matter using blood oxygen level-dependent (BOLD) signals in functional MRI (fMRI) has been technically and physiologically challenging (Gawryluk et al., 2014; Gore et al., 2019; Grajauskas et al., 2019). Historically, white matter signals in fMRI studies were treated as noise or nuisance variables, often excluded during preprocessing due to a long-standing focus on gray matter, which contains dense synaptic connections and plays a primary role in neural processing (Frackowiak et al., 2004; Gonzalez-Castillo et al., 2012; Grajauskas et al., 2019). However, recent advancements in analysis strategies and validation efforts have demonstrated that white matter BOLD signals can be reliably detected, aligned with cortical functional connectivity patterns, and linked to similar physiological properties with gray matter activity, even using standard fMRI acquisition protocols (Gao et al., 2020, 2021; Ding et al., 2013, 2016; Li et al., 2019; Schilling et al., 2023).

Building on these advances, research over the past decade has established the validity and reliability of measuring functional white matter properties. Several key findings support this approach. First, functional connectivity between white matter tracts and gray matter regions exhibits organized patterns that resemble structural connectivity observed in diffusion MRI (Ding et al., 2013; Huang et al., 2018; Zhao et al., 2022). Second, task performance enhances functional coupling between white matter tracts and cortical regions responsible for visual processing, motor functioning, working memory, and language processing (Ding et al., 2016, 2018; Gao et al., 2021; Huang et al., 2018; Schilling et al., 2023). Specifically, when participants engage in cognitively demanding tasks, increased BOLD signal synchronization emerges between relevant cortical areas and the white matter pathways that support those networks; for example, greater coupling between the occipital regions and optic radiations for visual tasks, and between the pre- and post-central sulci and corticospinal tracts for motor or sensory tasks (Schilling et al., 2023). These findings suggest that white matter tracts are not merely passive conduits but functionally involved in dynamic, task-relevant information transfer. Third, resting-state functional correlations within white matter tracts mirror structural pathways essential for efficient information transfer (Ding et al., 2013, 2016; Zhao et al., 2022). Fourth, reliable detection of BOLD signal fluctuations across major white matter pathways, including the uncinate fasciculus, has been demonstrated (Ding et al., 2016; Fabri et al., 2014; Schilling et al., 2023; Tettamanti et al., 2002). Finally, research on functional white matter has yielded novel insights into clinical populations, such as individuals with schizophrenia (Gao et al., 2020, 2021). Despite these advances, no studies to date have examined the functional interactions between white matter tracts and cortical regions in the context of reading development. Investigating such interactions could provide a crucial framework for understanding the role of white matter pathways in supporting higher-level linguistic functions.

Various white matter pathways have been linked to language processing, including the left arcuate, superior longitudinal, inferior longitudinal, inferior frontal-occipital, and uncinate fasciculi (Arrington et al., 2017; Del Tufo et al., 2019; Feldman et al., 2012; Giampiccolo & Duffau, 2022; Giampiccolo et al., 2025; Harvey et al., 2013; Lebel et al., 2019; Meisler & Gabrieli, 2022; Ng et al., 2021; Roy et al., 2024; Saygin et al., 2013; Vandermosten et al., 2012; Zemmoura et al., 2015). While the arcuate, inferior longitudinal, and inferior frontal-occipital fasciculi have been strongly implicated in phonological and orthographic processing during word reading, the uncinate fasciculus has been associated with higher-level semantic and integrative language functions relevant for comprehension (Catani & De Schotten, 2008; Dick et al., 2014; Friederici, 2011; Shekari & Nozari, 2023). The left uncinate fasciculus, a ventral frontal-temporal tract, is thought to support higher-level linguistic processes, such as semantic retrieval, integration, and control (Catani & De Schotten, 2008; Dick et al., 2014; Friederici, 2011; Shekari & Nozari, 2023). Clinical and lesion studies lend support to this role: damage to the uncinate fasciculus has been associated with impairments in semantic processing, including deficits in naming, verbal memory, and comprehension, particularly for tasks requiring controlled retrieval of meaning or integration of semantic context (Basilakos et al., 2014; Catani et al., 2013; Harvey et al., 2013; Meinzer et al., 2010; Papagno, 2011; Stefańska & Kieronska-Siwak, 2025). Individuals with lesions affecting the uncinate fasciculus often show difficulties in understanding complex language or in tasks involving semantic association, further implicating this pathway in controlled semantic access (Basilakos et al., 2014; Catani et al., 2013; Harvey et al., 2013; Meinzer et al., 2010; Papagno, 2011; Stefańska & Kieronska-Siwak, 2025). The uncinate fasciculus structurally connects the anterior ventral inferior frontal cortex with the anterior temporal lobe, enabling semantic information to be relayed through the temporal pole from posterior and medial temporal regions to the frontal lobe (Briggs et al., 2021; Catani & De Schotten, 2008; Friederici, 2011). The gray matter regions structurally linked to the uncinate fasciculus include anterior temporal and anterior medial temporal regions supporting semantic memory and representation, and ventrolateral prefrontal regions involved in semantic control (Hau et al., 2016, 2017; Leng et al., 2016; Olson et al., 2015; Panesar et al., 2017; Weiller et al., 2021). Together, these regions form a frontal-temporal semantic network that enables flexible, goal-directed access to meaning (Chiou et al., 2018; Jackson, 2021; Lambon Ralph et al., 2017; Qin et al., 2021). In the context of reading, this network supports skilled reading comprehension by coordinating the higher-level processes required to construct meaning from text (Aboud et al., 2016, 2019; Keller et al., 2024).

During reading comprehension, individual words must be processed and then integrated within and across sentences to build coherent meaning. As such, it follows that a core component of skilled reading comprehension is efficient word recognition, or ability to efficiently decode and identify written words (Castles et al., 2018; Hoover & Gough, 1990; Perfetti et al., 2005; Tunmer & Chapman, 2012). Word recognition engages brain regions such as the left occipital-temporal cortex and the supramarginal gyrus (Aboud et al., 2016; Burgess & Cutting, 2023; Church et al., 2008; Landi et al., 2013; Ryherd et al., 2018). Individuals who have weaker word recognition skills often are less proficient at reading comprehension (Perfetti et al., 2005). However, comprehension also depends on higher-level semantic and executive processes that allow readers to construct meaning from context and prior knowledge. Notably, some readers, especially those with weaker word recognition, may still achieve proficient comprehension by relying more heavily on these higher-level mechanisms (Aboud et al., 2018; Bailey et al., 2016; Cutting et al, 2013; Finn et al., 2014; Rimrodt et al, 2009). Prior research has shown that increased connectivity among regions involved in semantic memory and control, such as the left anterior temporal lobe, inferior frontal gyrus, and lateral prefrontal cortex, can help compensate for weak word recognition skills, providing a more meaning-driven route to comprehension (Aboud et al., 2018; Cutting et al., 2013; Farris et al., 2011; Finn et al., 2014; Lukic et al., 2025; Mateu-Estivill et al., 2021; Rimrodt et al., 2009). Thus, across the reading ability spectrum, readers may engage different neural strategies for understanding text, with some relying more on semantic pathways when word recognition is less efficient. Nevertheless, the precise neural mechanisms underlying this flexible or compensatory process, especially linked to white matter tracts that connect gray matter regions involved in higher-level linguistic processes, remain unclear.

The current study examines how functional connectivity between the left uncinate fasciculus and structurally linked gray matter regions involved in semantic memory and control modulates the relationship between word recognition and reading comprehension. We focus on the uncinate fasciculus because of its anatomical role in linking temporal and frontal semantic systems, forming a frontal-temporal semantic network. We hypothesize that stronger functional connectivity within this network will weaken the typical dependence of reading comprehension on word recognition skill, reflecting a flexible reading architecture that draws on semantic systems to support meaning making. Rather than framing semantic involvement as a compensatory response to word recognition difficulties, we explore its broader role as a dynamic contributor to reading comprehension across the full range of ability.

## 2 Methods

All procedures were approved by and carried out in accordance with Vanderbilt University’s Institutional Review Board. Data for the current analysis came from a larger overarching study focused on investigating the neural and behavioral bases of reading comprehension in a cross-section of adolescents (R01 HD044073). (For more information on this larger study, refer to Aboud et al., 2016; Miller et al., 2014; Spencer et al., 2020; Wilkey et al., 2018.)

Participants were recruited from local schools, clinics, and pediatricians’ offices, as well as the greater Nashville and Middle Tennessee communities, via flyers, bulletins, and the internet. Participants were between 10 and 14 years old at the time of enrollment. At the beginning of the study, a parent or guardian provided informed consent, and the participating child provided verbal assent. Participants received compensation for their participation in the study.

### 2.1 Participants

Participants were included in the larger study if they met the following criteria: native speakers of American English with normal hearing, normal or corrected-to-normal vision, no known history of major psychiatric or neurological problems or traumatic brain injury/epilepsy, no history of a major intellectual or developmental difficulty, and compatibility with MRI. Participants who met the inclusion criteria were invited to participate in 2 days of behavioral testing with trained graduate-level research assistants and/or staff members, followed by an optional MRI scanning session on the second day. Participants received compensation for their participation in the study. A total of 155 participants completed both behavioral testing and MRI scans, including T1-weighted structural MRI and resting-state functional MRI data.

All MRI data were examined for data quality. A total of 53 participants had high-quality MRI data that passed quality control measures for motion. Resting-state functional MRI data were retained if the maximal translations and rotations of head motion were less than 1.5 mm and 1.5^o^, respectively (see MRI data processing). The final sub-sample included 29 girls (55%) and 24 boys (45%) in this sample. Within this sub-sample, 2 participants identified as Asian, 3 participants identified as Black or African American, 37 participants identified as White, 7 identified as more than one race, and 4 did not report or selected “Prefer not to answer.” Participants in our sample came from families with parents who had on average completed some college. Handedness was evaluated using the *Edinburgh Handedness Inventory* (Veale, 2014).

### 2.2 Reading measures

#### 2.2.1 Word recognition skill

The Letter-Word Identification and Word Attack subtests from the *Woodcock-Johnson III* (WJ; Woodcock et al., 2001) were used to measure each participant’s accuracy in recognizing real words and decoding non-words, respectively (α’s = 0.91–0.94). Scores from these subtests made up the Basic Reading composite scores; age-normed standard scores for Basic Reading were used in our analysis to capture word recognition skill.

#### 2.2.2 Reading comprehension ability

The Passage Comprehension test from the *Woodcock-Johnson III* (Woodcock et al., 2001) was used to measure each participant’s ability to read a passage of text and fill in missing words within each passage (α = 0.88). Age-normed standard scores for Passage Comprehension were used in our analysis to capture reading comprehension ability.

### 2.3 Functional MRI measures

#### 2.3.1 MRI data acquisition

Brain imaging was performed at the Vanderbilt University Institute of Imaging Science. Prior to the MRI scan session, participants were introduced to the facility and visited a mock scanner at the Institute of Imaging Science, which allowed them to go through a practice scan, become familiarized with MRI scanner noise, and practice lying still in the machine. Resting-state functional MRI and structural MRI data were collected on a Philips Achieva 3 Tesla MR scanner with an eight-channel head coil. Scans were oriented along the anterior commissure (AC) and posterior commissure (PC) plane.

Resting-state functional T2*-weighted MRI scans were acquired using a gradient echo-echo planar imaging (GE-EPI) pulse sequence with the following parameters: 160 volumes; 40 axial slices; inter-slice gap of 1 mm; slice thickness of 3 mm; repetition time (TR) of 2200 milliseconds; echo time (TE) of 30 milliseconds; flip angle of 75^o^; voxel size of 1.875 x 1.875 x 3 mm^3^; acquisition time of 365.2 seconds; field of view of 240 x 240 mm^2^; imaging matrix of 128 x 128. To allow for steady-state magnetization to be reached before acquiring the functional data, five dummy volumes were added at the beginning, which were subsequently removed from analysis. Participants were instructed to keep their eyes open, try to remain awake and still, and look at a fixation cross presented in the center of the visual field.

Structural T1-weighted MRI scans were acquired using the Magnetization Prepared Rapid Gradient Recalled Echo sequence (MP-RAGE; Mugler & Brookeman, 1990) according to the following parameters: 256 x 256 in plane resolution; 170 sagittal slices with no inter-slice gap; slice thickness of 1 mm; repetition time (TR) of 8.1 milliseconds; echo time (TE) of 3.7 milliseconds; flip angle of 7^o^; voxel size of 1 mm^3^ isotropic; acquisition time of 392 seconds. Participants were instructed to try to remain still while watching a movie.

#### 2.3.2 MRI data processing

MRI data were processed using an automated high-performance pipeline as extensively described by Gao et al. (2021, 2023). Data processing was done through the XNAT environment hosted by the Vanderbilt University Institute of Imaging Science (Harrigan et al., 2016). The processing pipeline was established based on the Data Processing and Analysis for Brain Imaging (DPABI) toolbox, which operates in the Montreal Neurological Institute (MNI) space defined by ICBM152 nonlinear asymmetric template (MNI152NLin2009cAsym). The Johns Hopkins University ICBM-DTI-81 atlas, which is based on the MNI152NLin6Asym template, was used to define white matter regions. We co-registered the MNI152NLin6Asym template to the MNI152NLin2009cAsym template and applied the resulting transformation field to the ICBM-DTI-81 atlas. Raw resting-state functional MRI data were corrected for slice timing and head motion. We then regressed out 24 motion-related parameters and the mean cerebrospinal fluid signal to minimize the influence of movement and physiological noise. The data were detrended and temporally filtered with a passband frequency of 0.01–0.1 Hz. Tissue probability maps for gray matter, white matter, and cerebrospinal fluid were generated through segmentation of structural MRI data. The preprocessed functional MRI data and tissue probability maps were subsequently spatially normalized into MNI space using co-registration and normalization procedures. To mitigate partial volume effects between white and gray matter, spatial normalization of fMRI data to MNI space was performed using nearest neighbor interpolation, and no spatial smoothing was applied.

After preprocessing, data quality control was performed. Datasets were retained if spatial normalization was acceptable and maximal head motion did not exceed 1.5 mm of translation or 1.5^o^ of rotation (Cantlon & Li, 2013; Chantiluke et al., 2012; Rocca et al., 2013; Rubia et al., 2009; Wang et al., 2017; Yin et al., 2021). This threshold was chosen to balance data retention with signal reliability, particularly given the short resting-state run (~6 minutes) and the greater susceptibility of white matter signals to motion-related artifacts and partial volume effects. To account for residual motion effects, mean frame-wise displacement (FD; *m* = 0.17, *sd* = 0.04) was also included as a covariate in analysis.

#### 2.3.3 Functional connectivity

The white matter bundles were defined using the Johns Hopkins University’s *ICBM-DTI-81 Eve* white matter atlas (Oishi et al., 2009; Mori et al., 2008), which includes 48 white matter bundles, while gray matter parcels were defined according to the *PickAtlas Brodmann* gray matter atlas (Lancaster et al., 2000), which includes 82 gray matter areas. To prevent signal contamination to adjacent white and gray matter, individual tissue probability maps were thresholded at 0.80 to create subject-specific white matter and gray matter masks.

White matter–gray matter resting-state functional connectivity was estimated by calculating Pearson’s correlation coefficients between regional functional MRI time series from predefined white matter bundles (with a focus on the left uncinate fasciculus) and gray matter parcels in MNI space.

#### 2.3.4 White matter tract of interest

For each participant, correlation coefficients between BOLD signals in the left uncinate fasciculus and 82 gray matter regions were extracted to analyze associations with reading scores. Given prior functional brain connectivity findings that highlight the role of the inferior frontal-anterior temporal language network in word and passage reading performance (e.g., Aboud et al., 2016), we examined connectivity patterns between the left uncinate fasciculus bundle and gray matter structures.

#### 2.3.5 Gray matter regions of interest

For each participant, correlation coefficients between BOLD signals in the left uncinate fasciculus and gray matter regions were extracted, focusing on gray matter regions known to be structurally linked with the left uncinate fasciculus. Based on prior anatomical studies (Briggs et al., 2019; Hau et al., 2016, 2017; Leng et al., 2016; Olson et al., 2015; Panesar et al., 2017; Weiller et al., 2021), these regions of interest included the anterior temporal and anterior medial temporal regions (BA 38, 36, 35), which support semantic memory and representation, as well as areas in the medial and orbitofrontal cortex (BA 25, 10, 11) and the ventral inferior frontal gyrus (BA 47, 45), which are implicated in control and regulatory processes.

#### 2.3.6 White matter tracts for control comparisons

To contextualize the specificity of findings related to the left uncinate fasciculus, control analyses were conducted to examine functional connectivity patterns of other tracts in relation to reading scores for comparison. These control tracts included the right uncinate fasciculus, left sagittal stratum, and left cingulum gyrus. Each was chosen for its anatomical relevance to reading and its distinct functional profile, providing a framework to assess whether observed effects were unique to the left uncinate fasciculus or reflective of broader white matter–gray matter connectivity patterns. The right uncinate fasciculus was included to test hemispheric specificity. Although structurally homologous to the left uncinate fasciculus, the right uncinate fasciculus is not typically implicated in language or semantic processing to the same extent, and it has been more commonly associated with socio-emotional and affective processing (Shekari & Nozari, 2023). Comparing left versus right uncinate fasciculus connectivity allowed us to evaluate whether the observed reading-related patterns were lateralized and functionally specific. The left sagittal stratum—a composite tract encompassing the inferior longitudinal fasciculus and inferior frontal-occipital fasciculus—was selected because these tracts have been shown in prior diffusion MRI studies to participate in the ventral reading stream and support reading-related processes (Del Tufo et al., 2019; Lebel et al., 2013; Meisler et al., 2024). Including this tract helps determine whether functional connectivity findings attributed to the uncinate fasciculus are distinct or shared across neighboring ventral pathways. Finally, the left cingulum bundle was selected chosen as a control tract not directly implicated in the ventral reading stream. The cingulum supports internally directed cognitive processes such as autobiographical memory and cognitive control (Gao et al., 2020). Its inclusion provides a baseline for examining whether reading-related functional connectivity patterns are specific to language-relevant white matter pathways.

### 2.4 Statistical analysis

Analyses were conducted in *R* (Version 4.3.0) to examine associations between reading scores and white- and gray-matter functional connectivity, with a focus on the left uncinate fasciculus and its structurally linked gray matter regions. Correlation between reading measures was computed using scores on the WJ Basic Reading and Passage Comprehension tests to assess their association.

**Fig. 1.**
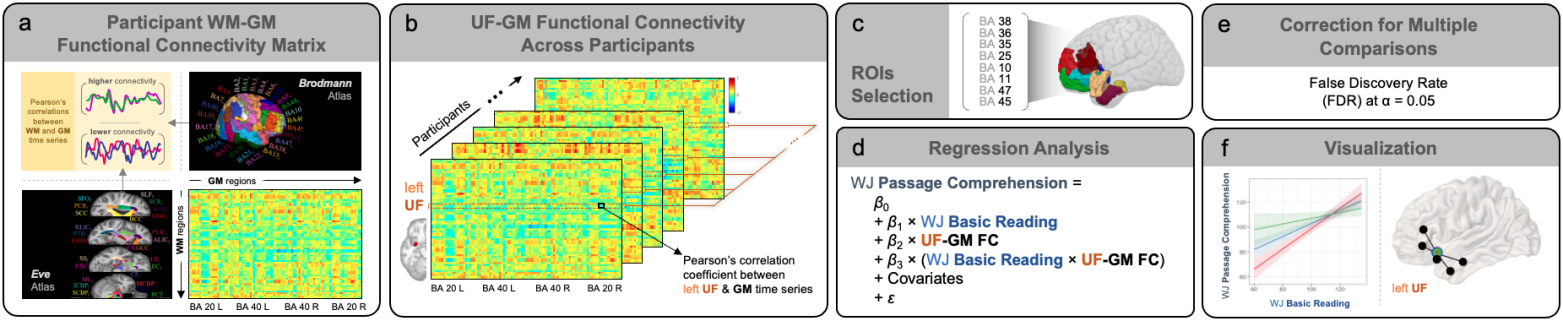
Visualization of the analysis pipeline. (a) Pearson’s correlations between white matter (WM) and gray matter (GM) time series were computed to create each participant’s functional connectivity matrix, with rows representing 48 WM bundles from the Eve atlas and columns representing 82 GM regions from the Brodmann’s Areas (BA) atlas. Reproduced with permission from Gao et al. (2021). (b) Functional connectivity indices between the uncinate fasciculus (UF) and GM regions were derived from the WM-GM functional connectivity matrices across participants. (c) Functional connectivity indices with the uncinate fasciculus were filtered by structurally linked GM regions of interest (ROIs). (d) Regression analysis was conducted to examine differences in WJ Passage Comprehension scores related to the interaction between WJ Basic Reading scores and UF-GM ROI functional connectivity (FC). (e) Multiple comparisons were corrected using False Discovery Rate (FDR). (f) Results surviving multiple comparison correction, including significant interaction effects and UF-GM ROI functional connectivity patterns, were visualized.

Figure 1 includes a visualization of the analysis pipeline following the calculation of Pearson’s correlations between regional functional MRI time series to estimate white matter–gray matter functional connectivity. Functional connectivity indices between the left uncinate fasciculus and gray matter regions were extracted from white matter and gray matter functional connectivity matrices across participants. To interrogate associations between functional connectivity of the left uncinate fasciculus bundle and reading performance, we conducted regression analyses. Functional connectivity indices were derived from white and gray matter connectivity data and correlated with reading scores. Functional connectivity analysis focused indices between the uncinate fasciculus and structurally linked gray matter regions. Scores on the WJ Passage Comprehension test served as the dependent variable, with WJ Basic Reading scores and functional connectivity metrics entered as predictors. Specifically, we modeled an interaction effect between WJ Basic Reading scores and uncinate fasciculus functional connectivity metrics to predict Passage Comprehension scores.

The primary analysis evaluated whether functional connectivity between the uncinate fasciculus and structurally linked gray matter regions moderated the relationship between word recognition and reading comprehension. To do so, linear regression models were estimated in which WJ Passage Comprehension scores were predicted by WJ Basic Reading scores (as an index of word recognition), uncinate fasciculus–gray matter functional connectivity coefficients, and their interaction term. Statistically, the inclusion of the interaction term allowed us to test whether the strength and/or direction of the association between word recognition and reading comprehension varied as a function of white matter–gray matter connectivity. This models the hypothesis that functional coupling between the uncinate fasciculus and semantic memory and control regions may modulate reading outcomes differently depending on a child’s word recognition ability, potentially providing compensatory or flexible support when word recognition is insufficient. Analysis accounted for confounding factors, including sex, age, handedness, and head motion (quantified using mean FD). False discovery rate (FDR) correction was applied across eight regressions (eight gray matter regions of interest) to adjust for multiple comparisons, using a significance threshold of α = 0.05. For all regions where the interaction term remained significant after correction, interaction plots were generated to visualize how the relationship between word recognition and reading comprehension varied by uncinate fasciculus functional connectivity strength.

To further assess the specificity of findings for the left uncinate fasciculus, we conducted supplemental analyses. Here, we repeated the regression models to examine interactions between reading scores and functional connectivity patterns in additional white matter tracts, selected as control comparisons. These tracts included the right uncinate fasciculus, left sagittal stratum, and left cingulum gyrus. This supplemental analysis allowed us to explore whether connectivity patterns in these regions similarly influenced the association between gray matter structures and reading scores.

## 3 Results

### 3.1 Correlation between word recognition and reading comprehension

**Fig. 2.**
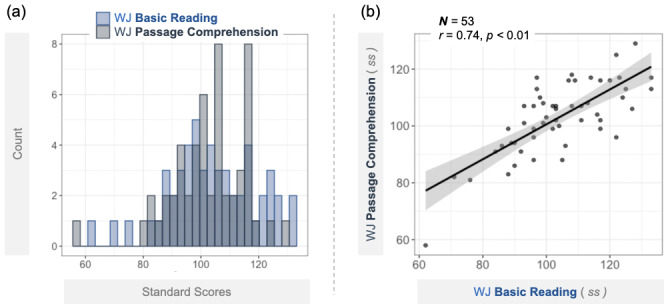
(a) Distributions of reading scores measured by WJ Basic Reading and Passage Comprehension. (b) Correlation between WJ Basic Reading and Passage Comprehension scores. (*Note*. ss = standard scores.)

Descriptive statistics for the participant sample (*N* = 53) are presented in Table 1. The distribution of reading scores from WJ Basic Reading and Passage Comprehension, along with their correlation plot, is presented in Figure 2. The WJ Basic Reading measure had a *mean* score of 103.94 (*sd* = 15.34, *range* = 62–133), while the WJ Passage Comprehension measure had a *mean* score of 102.98 (*sd* = 12.66, *range* = 58–129). Consistent with expectations that word recognition is foundational to reading comprehension (e.g., Perfetti et al., 2005), a significant positive correlation was observed between WJ Basic Reading and Passage Comprehension scores (*r* = 0.74, *p* < 0.01).

**Table 1.** Descriptive statistics for our participant sample (*N* = 53).

(*N* = 53)	*m*	*sd*	*range*
Age	11.57	1.33	[10, 14]
Sex	29 girls, 24 boys		
Handedness (Raw Scores)	66.66	35.83	[-80, 100]
WJ Basic Reading (Standard Scores)	103.94	15.34	[62, 133]
WJ Passage Comprehension (Standard Scores)	102.98	12.66	[58, 129]

### 3.2 Functional connectivity between white matter and gray matter structures

In a pediatric sample, we replicated prior findings on white matter functional connectivity patterns in mature or clinical populations (Gao et al., 2020, 2021; Ding et al., 2018; Zu et al., 2024). Supplementary Figure S1 presents the average resting-state temporal correlations between BOLD signals in 48 white matter bundles (from the Eve atlas) and 82 gray matter regions (from the Brodmann atlas), where each cell represents a mean correlation coefficient across participants (*N* = 53). The results are consistent with previous research showing that specific white matter bundles, including the cingulum, corpus callosum, external capsules, and internal capsules, tend to exhibit higher mean connectivity with specific gray matter regions (Gao et al., 2020, 2021; Ding et al., 2018; Zu et al., 2024). These connectivity patterns, displayed as horizontal “stripes,” suggest synchronous activity across particular white and gray matter regions, indicating organized resting-state connectivity patterns within the brain.

### 3.3 Moderation of reading performance by uncinate fasciculus functional connectivity

**Fig. 3.**
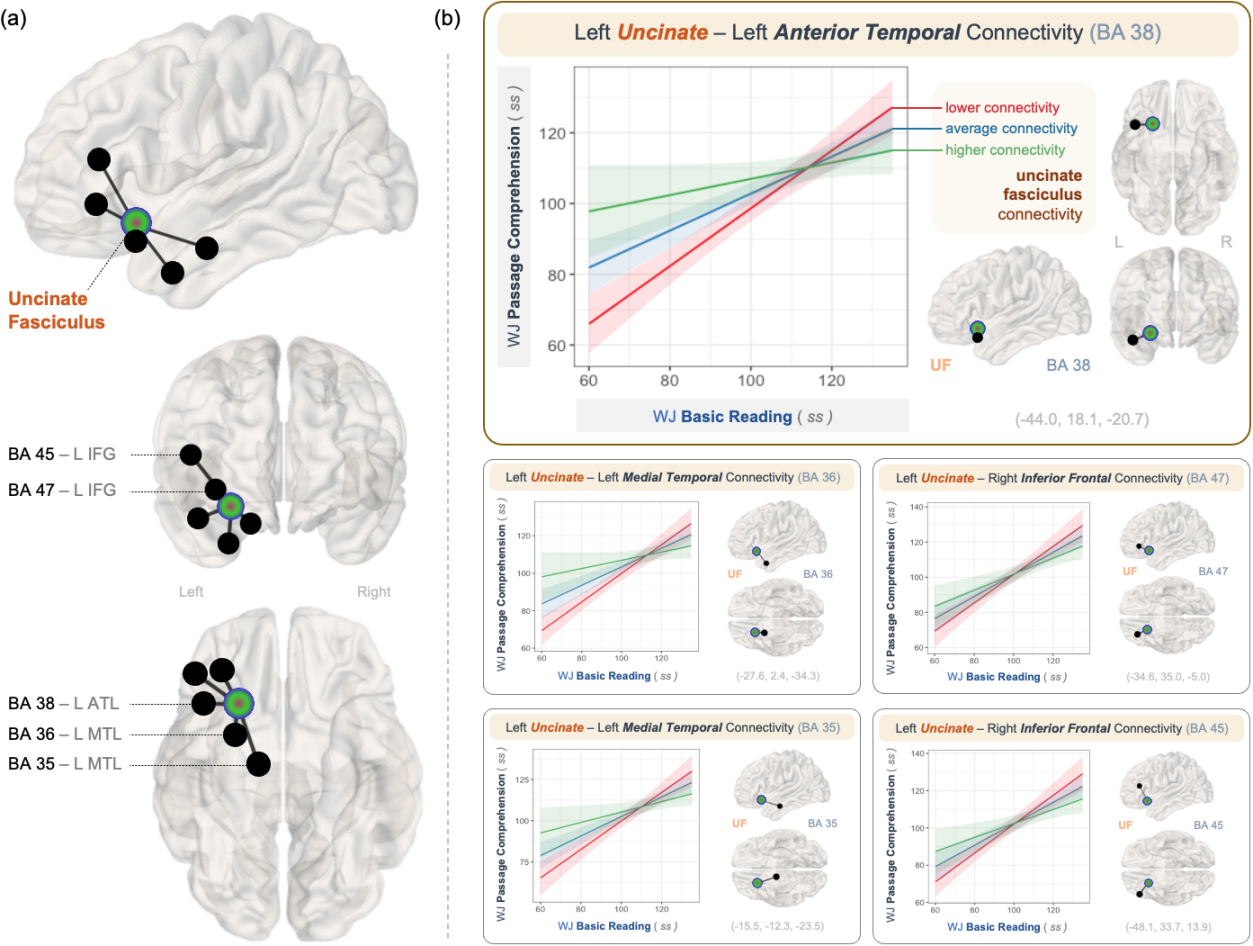
(a) Visualization of the left uncinate fasciculus (UF) functional connectivity with structurally linked gray matter structures that showed significant interaction effects with reading scores after correcting for multiple comparisons with FDR at α = 0.05. (b) Interaction plots of the associations between reading scores varying functional connectivity between the left uncinate fasciculus and the left anterior temporal lobe (ATL; BA 38), anterior medial temporal lobe (MTL; BA 35, 36), and inferior frontal gyrus (IFG; BA 45, 47) (reported in Table 2). (*Note*. *ss* = standard scores.)

After correcting for multiple comparisons, significant interaction effects emerged between WJ Basic Reading scores and functional connectivity between the left uncinate fasciculus and multiple gray matter regions in predicting WJ Passage Comprehension scores (Table 2; Fig. 3). Specifically, the strength of association between WJ Basic Reading and Passage Comprehension scores varied by the level of functional connectivity between the left uncinate fasciculus and several gray matter structures: left anterior temporal lobe (ATL; BA 38; *b* = -0.350, *se* = 0.090), left medial temporal regions (MTL; BA 36, *b* = -0.326, *se* = 0.086; and BA 35, *b* = -0.326, *se* = 0.120), and left inferior frontal regions (IFG; BA 47, *b* = -0.204, *se* = 0.089; and BA 45, *b* = -0.238, *se* = 0.082). All interaction terms remained significant after false discovery rate (FDR) correction (*p* < 0.05).

**Table 2.** Left uncinate fasciculus and structurally connected gray matter functional connectivity interaction analysis with reading scores (WJ Basic Reading and Passage Comprehension measures).

White matter bundle	Gray matter regions	MNI Coordinates	*b*	*se*	raw *p*	FDR *p*
Left uncinate fasciculus	L BA 38: Left anterior temporal lobe (ATL)	(-44, 18, -21)	-0.350	0.090	0.000	0.002
	L BA 36: Left medial temporal lobe (MTL)	(-28, 2, -34)	-0.326	0.086	0.000	0.002
	L BA 35: Left medial temporal lobe (MTL)	(-16, -12, -24)	-0.326	0.120	0.009	0.019
	L BA 25: Left subgenual area	(-7, 16, -5)	-0.011	0.108	0.919	0.919
	L BA 10: Left anterior prefrontal cortex	(-15, 59, 10)	-0.163	0.080	0.047	0.062
	L BA 11: Left orbitofrontal cortex	(-15, 42, -12)	-0.180	0.107	0.098	0.112
	L BA 47: Left inferior frontal gyrus (IFG)	(-35, 35, -5)	-0.204	0.089	0.026	0.042
	L BA 45: Left inferior frontal gyrus (IFG)	(-48, 34, 14)	-0.238	0.082	0.005	0.014

Connection pairs reported had *p* values surviving correction for multiple comparisons with FDR at α = 0.05.

Visualization of the interaction effects (Fig. 3b) demonstrated that functional connectivity between the left uncinate fasciculus and its structurally connected gray matter regions modulated how strongly word recognition predicted reading comprehension. When uncinate connectivity, particularly with the ATL (BA 38), is low, comprehension is tightly linked to word recognition skill: children with weaker word recognition showed significantly lower comprehension, but performance improved steeply as word recognition strengthened. In contrast, higher uncinate–ATL connectivity was associated with relatively strong comprehension across all levels of word recognition, including for readers with weaker word recognition.

Similar moderation effects emerged for uncinate connectivity with the MTL (BA 36, 35) and IFG (BA 47, 45), regions involved in semantic memory and control. These patterns suggest that the uncinate fasciculus could support a flexible reading system, enabling readers to draw on meaning-based strategies when phonological decoding is less reliable. Notably, this decoupling between word recognition and comprehension was observed across the full range of abilities, highlighting semantic scaffolding as a general mechanism—not just a compensatory one in struggling readers. Stronger connectivity within this frontal-temporal semantic network may allow children to shift from decoding-driven to meaning-driven reading, depending on their skill profile and task demands. When frontal-temporal semantic regions are more functionally connected through the uncinate, children may be better equipped to extract meaning from text using context and prior knowledge, even in the presence of word recognition difficulties.

Other structurally connected regions in the left medial and orbitofrontal cortex (BA 10, 11, 15) did not exhibit significant interaction effects, highlighting the selectivity of the uncinate fasciculus’s functional role in modulating decoding–comprehension relationship through the semantic network.

### 3.4 Supplemental analysis with alternative white matter tracts as control comparisons

To contextualize the left uncinate fasciculus findings, interaction analyses were repeated using functional connectivity indices from the right uncinate fasciculus, left sagittal stratum, and left cingulum gyrus. No significant interactions were detected between these tracts and gray matter structures with respect to reading scores, as none of the tested connection pairs survived correction for multiple comparisons at α = 0.05. These limited effects further support the role of the left uncinate fasciculus in moderating the relationship between word recognition skill and reading comprehension ability.

## 4 Discussion

This study investigated how functional connectivity between the left uncinate fasciculus, a ventral white matter tract linking anterior temporal and inferior frontal language regions, and structurally connected gray matter regions modulates the relationship between word recognition and reading comprehension in adolescent readers. Specifically, we tested whether functional coupling between the uncinate and gray matter regions involved in semantic representation and memory (anterior and medial temporal lobes) and semantic control (inferior frontal gyrus) altered the extent to which comprehension depends on word recognition skill. Our findings showed that uncinate fasciculus functional connectivity with the frontal-temporal semantic gray matter regions moderated the strength of the association between word recognition and reading comprehension across the full range of reading ability. Among participants with lower uncinate connectivity, reading comprehension was strongly dependent on word recognition; children with weaker word recognition showed markedly lower comprehension, but comprehension improved sharpened with word recognition skill. In contrast, among those with higher uncinate connectivity, comprehension was more consistent across levels of word recognition, including for readers with weaker word recognition. This pattern indicates a decoupling of decoding and comprehension, suggesting that increased functional engagement of the frontal-temporal semantic network enables readers to rely less on decoding and more on meaning-based processing. Crucially, this moderating effect was observed not only among readers with word recognition difficulties, but also across the entire skill distribution, highlighting the flexible role of the uncinate fasciculus in supporting comprehension. These findings support the notion of flexible semantic scaffolding, in which stronger connectivity through the uncinate may enable readers’ access to semantic content independent of word recognition skill.

These results on frontal-temporal semantic network connectivity with the uncinate fasciculus align with previous fMRI work implicating the left anterior temporal lobe (ATL; BA 38) as a key hub of the brain’s semantic work, responsible for integrating multi-modal conceptual information and meaning making (Aboud et al., 2016, 2023; Lambon Ralph et al., 2017; Patterson et al., 2007; Simmons et al., 2010; Visser et al., 2012). The anterior medial temporal lobe (MTL; BA 35 and 36) is similarly implicated in semantic memory and lexical representations, particularly through its tight functional and structural connections with the ATL (Ghetti & Bunge, 2012; Maguire et al., 2000; Raud et al., 2023). Strong uncinate fasciculus connectivity with these temporal regions may facilitate access to semantic knowledge that supports comprehension, regardless of word recognition proficiency. We also found that functional coupling between the uncinate and the ventral inferior frontal gyrus (IFG; BA 45 and 47) moderated the decoding–comprehension relationship. The IFG is implicated in semantic control, syntactic processing, and flexible engagement with orthographic-semantic and discourse-level networks (Aboud et al., 2016; Chiou et al., 2018; Fedorenko et al., 2012; Hagoort & Indefrey, 2014; Jackson et al., 2021). The IFG’s role in multiple-demand and executive control networks may further enable flexible semantic retrieval and integration under varying task demands (Demeter et al., 2023; Duncan, 2010; Engelhardt et al., 2019; Fedorenko et al., 2012). The moderating effect observed for IFG connectivity suggests that stronger functional coupling supports flexible semantic retrieval and top-down control, which may reduce reliance on phonological decoding during comprehension across diverse reading profiles. Put together, our findings suggest that when these frontal-temporal systems are functionally coupled with the uncinate, children may draw more heavily on meaning-based processing to support comprehension, regardless of their word recognition proficiency.

These functionally connected systems align with known anatomical pathways: uncinate fasciculus structurally links anterior temporal regions (BA 35, 36, and 38) with ventral inferior frontal regions (BA 47 and 45), forming a critical component of the ventral language stream implicated in sematic retrieval, integration, and control (Briggs et al., 2019; Catani & De Schotten, 2008; Dick et al., 2014; Friederici, 2011; Hau et al., 2016, 2017; Leng et al., 2016; Olson et al., 2015; Panesar et al., 2017; Shekari & Nozari, 2023; Weiller et al., 2021). By capturing resting-state BOLD signals in both white and gray matter, our findings show that this structural pathway is functionally engaged and may enable more flexible, meaning-based comprehension strategies. These results also extend theoretical models of reading that posit multiple processing pathways, including phonological and semantic, for accessing meaning (Coltheart et al., 2001; Seidenberg & McClelland, 1989). While semantic engagement is often framed as compensatory in struggling readers (Aboud et al., 2018; Cutting et al., 2013; Farris et al., 2016; Hoeft, 2011; Lukic et al., 2025), our findings suggest that stronger uncinate connectivity could facilitate meaning-based reading strategy, enabling readers to rely more on semantic systems and less on word recognition and decoding. This flexibility may reflect an adaptive property of the reading network, allowing readers to draw on available cognitive and neural resources to support comprehension in line with task demands and skill profiles.

Using recently developed methods to analyze white matter BOLD signals, the current study contributes evidence that functional interactions between white matter tracts and cortical regions can be detected at rest and are meaningfully related to individual differences in reading outcomes. These results underscore the value of incorporating white matter functional properties alongside gray matter measures of cognitive processes, particularly for reading. Future studies with task-based fMRI could further clarify the dynamic interactions between white and gray matter during reading and associations with reading outcomes (Ding et al., 2016, 2018; Meisler et al., 2024). Additionally, probing multiple fasciculi using dynamic causal modeling may offer further insights into how information is transferred across the cortex and among various white matter pathways. This study used a standard atlas-based definition of the uncinate fasciculus (Mori et al., 2008; Oishi et al., 2009), rather than representing the entire pathway. While this approach enables examination of functional correlations in white matter regions that are typically interpreted as the major white matter pathway associated with that region, given issues with crossing fibers and overlapping pathways across voxels (Schilling et al., 2022, 2025), the use of an atlas with non-overlapping tracts may introduce bias or limit anatomical specificity at the voxel size used. Future work may benefit from subject-specific tractography or use of more recent atlases such as Yeh (2022)’s HCP1065 to improve anatomical precision and account for partial volume effects, particularly in regions where white matter pathways overlap (Schilling et al., 2022, 2025). The current findings thus provide a foundation for understanding the integrated nature of neural networks in linguistic processing by capturing activity in white matter pathways, and further contribute to a growing body of research into the neural correlates of reading proficiency and difficulty.

In summary, this study shows that functional connectivity between the uncinate fasciculus and core semantic regions modulates the link between word recognition and reading comprehension across the reading ability spectrum. Stronger connectivity is associated with reduced dependence on word recognition, supporting a flexible architecture for comprehension that leverages semantic memory and control networks. These findings contribute to our growing understanding of the neural mechanisms supporting reading and suggest that white matter functional connectivity, particularly through the uncinate fasciculus, plays a critical role in supporting how readers of varying skill levels make meaning from text.

## Supplementary Material

Supplementary Material

## Data Availability

The data used for this study are not publicly available due to not all participants providing consent for their data to be shared outside of Vanderbilt University or Vanderbilt University Medical Center. A subset of these data, from those participants who did provide consent for their data to be shared outside of the aforementioned institutions, may be made available upon reasonable request to the corresponding author (LEC). MRI data processing pipeline and script can be accessed here: https://github.com/VUIIS/SCZ-WM-pipeline?organization=VUIIS&organization=VUIIS. All statistical analyses were conducted using the R programming language (R Core Team, 2023). Analysis script can be accessed here: https://github.com/EBRL/ImagingNeuroscience_ReadingWhiteGrayMatterfMRI.
