# Supplementary material for: Functional connectivity between the uncinate fasciculus and frontotemporal semantic system supports reading comprehension in adolescents
Source: Imaging Neurosci (Camb). 2025 Dec 12;3:IMAG.a.1055. doi: 10.1162/IMAG.a.1055 (PMC12703377; doi:10.1162/IMAG.a.1055)

SUPPLEMENTAL FIGURE

Supplemental Figure 1. Group-averaged white- and gray-matter functional connectivity (N = 53).

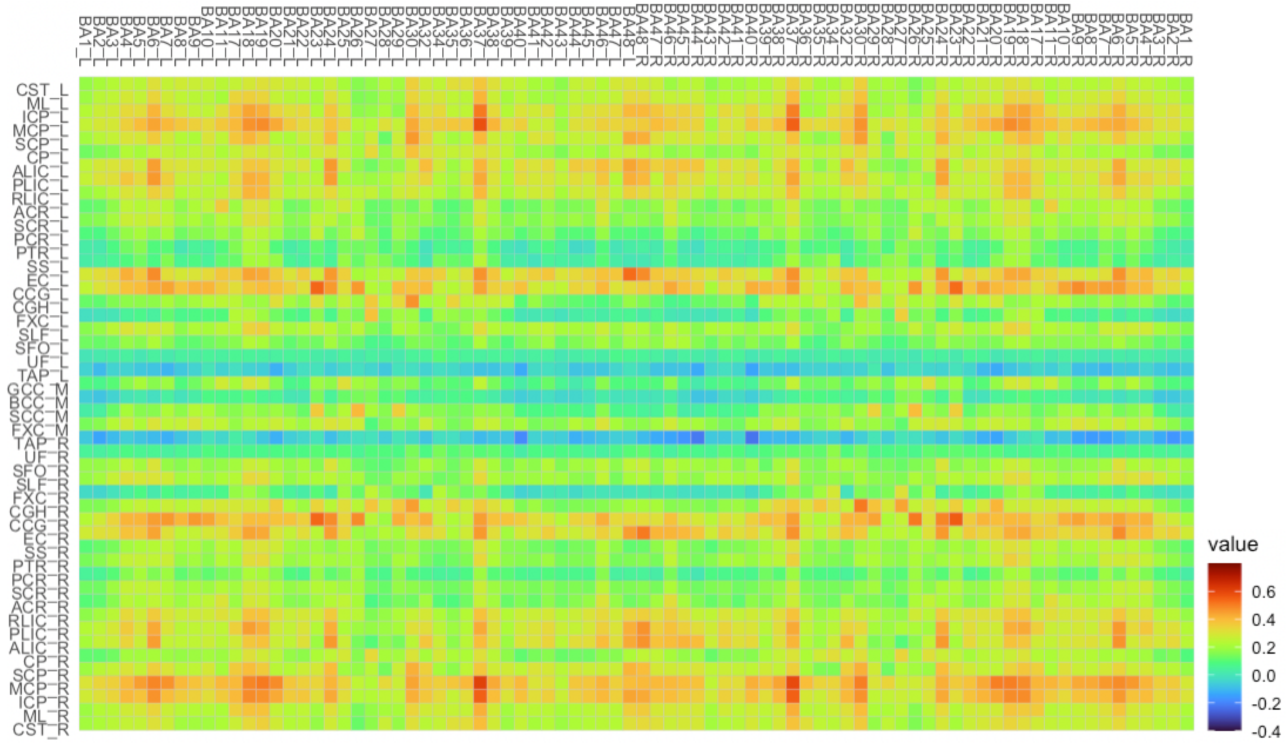

Supplement: Supplementary Material [file IMAG.a.1055_supp.pdf]
